# Assessment of GF-3 Polarimetric SAR Data for Physical Scattering Mechanism Analysis and Terrain Classification

**DOI:** 10.3390/s17122785

**Published:** 2017-12-01

**Authors:** Junjun Yin, Jian Yang, Qingjun Zhang

**Affiliations:** 1 The School of Computer and Communication Engineering, University of Science and Technology Beijing, Beijing 100083, China; yinjj07@gmail.com; 2 Department of Electronic Engineering, Tsinghua University, Beijing 100084, China; yangjian_ee@tsinghua.edu.cn; 3 Beijing Institute of Space System Engineering, China Academy of Space Technology, Beijing 100086, China

**Keywords:** GF-3 satellite, radar polarimetry, synthetic aperture radar (SAR), physical scattering mechanism

## Abstract

On 10 August 2016 China launched the GF-3, its first C-band polarimetric synthetic aperture radar (SAR) satellite, which was put into operation at the end of January, 2017. GF-3 polarimetric SAR has many advantages such as high resolution and multi-polarization imaging capabilities. Polarimetric SAR can fully characterize the backscatter property of targets, and thus it is of great interest to explore the physical scattering mechanisms of terrain types, which is very important in interpreting polarimetric SAR imagery and for its further usages in Earth observations. In this paper, focusing on target scattering characterization and feature extraction, we generalize the $\Delta \alpha_{B}/\alpha_{B}$ method, which was proposed under the reflection symmetric assumption, for the general backscatter process to account for both the reflection symmetry and asymmetry cases. Then, we evaluate the performances of physical scattering mechanism analysis methods for GF-3 polarimetric SAR imagery. Radarsat-2 data acquired over the same area is used for cross validation. Results show that GF-3 polarimetric SAR data has great potential for target characterization, especially for ocean area observation.

## 1. Introduction

Synthetic aperture radar (SAR) has all-weather, day and night imaging capabilities. In the past twenty years, a number of space-borne SAR systems designed for various Earth observation missions have been launched into Earth orbit. Typical polarimetric SAR systems include C-band RADARSAT-2, C-band Sentinel-1, L-band ALOS-2/PALSAR, X-band TerraSAR-X/TanDem-X, and the X-band COSMO-SkyMed satellite constellation. In August 2016, China launched the GF-3 satellite, its first polarimetric SAR satellite, which was in operation since January 2017. The GF-3 satellite has on-board a C-band high resolution SAR. It can work in twelve different imaging modes, in which the highest resolution is up to 1 m. It can provide fully polarimetric measurements with incidence angle ranging from 20° to 41°, and the antenna look direction can be either right or left. Nowadays, more and more polarimetric SAR satellites are going to be launched and planed. Efficient usage of these polarimetric SAR images is becoming a crucial problem.

Only a few studies on the use of GF-3 polarimetric SAR data for Earth observation applications can be found in the open literature. Wang et al. [1] used the rational polynomial coefficient model to study the geometric accuracy of GF-3 imagery. Yang et al. [2] investigated the application of GF-3 data for extracting information of ocean internal waves. Pan et al. [3] showed that GF-3 data is effective for fast vessel detection. Wang et al. [4] combined the parameters from Cloude-Pottier’s decomposition with the convolutional network for GF-3 image classification. The application performance of GF-3 polarimetric SAR data still needs to be fully assesses.

Polarimetric SAR can fully characterize the backscattering property of targets by using two orthogonal polarization channels, which allows development of different scattering models for different kinds of scatterers. Polarimetric feature extraction is fundamental for polarimetric target interpretation. Many parameter retrieval techniques and target decomposition methods [5,6,7,8,9,10,11,12] in terms of both coherent and incoherent categories have been developed for target feature extraction. Among the decomposition methods, the Cloude-Pottier decomposition provides a simple but effective method to identify the target scattering mechanisms. This method has been investigated and used widely in many applications. Yamaguchi’s four component decomposition [10] divides the backscattered energy into four different scattering mechanisms based on four scattering models and is widely used for polarimetric SAR image interpretation. In [13], based on the co-polarization ratio, we proposed a new physical scattering mechanism classification method to explore the intrinsic relationship between a single scattering mechanism and the stochastic backscattering process. This method was proposed based on the assumption of reflection symmetry, which is usually valid for natural scatterers, while for urban areas, backscatter can be both reflection symmetric and asymmetric due to the complexity of urban structures and alignments. In this paper, we further analyze the method presented in [13] under the reflection asymmetry condition. Then, together with Cloude-Pottier’s decomposition [6,14], this method is applied to analyze the performance of GF-3 polarimetric SAR data for representing target physical scattering mechanisms and terrain type classification capability.

The study is organized as follows: in Section 2, the method is introduced and analyzed for both reflection symmetry and asymmetry cases. In Section 3, by using both GF-3 polarimetric SAR data and Radarsat-2 polarimetric data collected over San Francisco area in the USA, several experiments are carried out. Conclusions are drawn in Section 4.

## 2. The Co-Polarization Ratio-Based Parameters and the $\Delta \alpha_{B}/\alpha_{B}$ Diagram

### 2.1. Parameters in the Reflection Symmetric Case

For reflection symmetric scatterers, an arbitrary backscattering coherency matrix can be expressed as follows [14]:

(1) $$T={\vec{k}}_{p}{\vec{k}}_{p}^{H}=\left[\begin{array}{ccc} T_{11} & T_{12} & T_{13}\\ T_{12}^{*} & T_{22} & T_{23}\\ T_{13}^{*} & T_{23}^{*} & T_{33} \end{array}\right]=Q{\left(2\theta \right)}^{T}\left[\begin{array}{ccc} t_{11} & t_{12} & 0\\ t_{12}^{*} & t_{22} & 0\\ 0 & 0 & t_{33} \end{array}\right]Q\left(2\theta \right),$$

where:

$$\{\begin{array}{c} t_{11}=\langle {\left|h+v\right|}^{2}\rangle \\ t_{22}+t_{33}=\langle {\left|h-v\right|}^{2}\rangle \\ t_{12}=\langle \left(h+v\right){\left(h-v\right)}^{*}\rangle \end{array};\;Q\left(2\theta \right)=\left[\begin{array}{ccc} 1 & 0 & 0\\ 0 & \cos\;2\theta & \sin\;2\theta \\ 0 & -\sin\;2\theta & \cos\;2\theta \end{array}\right].$$

h and v are the con-diagonalization parameters, ${\vec{k}}_{p}=\frac{1}{\sqrt{2}}{\left[\begin{array}{ccc} S_{\mathrm{HH}}+S_{\mathrm{VV}} & S_{\mathrm{HH}}-S_{\mathrm{VV}} & 2S_{\mathrm{HV}} \end{array}\right]}^{T}$ is the Pauli-basis vector; and 〈⋯〉 denotes ensemble averaging. From (1), a parameter $\alpha_{B}$ can be derived, which is a rotation invariant [13], as follows.

(2) $$\alpha_{B}=\tan^{-1}\left(\frac{T_{22}+T_{33}}{T_{11}}\right)=\tan^{-1}\left(\frac{{\left|\rho_{r}-1\right|}^{2}+2\left|\rho_{r}\right|\cos\;\phi_{r}\left(1-\left|r_{c}\right|\right)}{{\left|\rho_{r}+1\right|}^{2}-2\left|\rho_{r}\right|\cos\;\phi_{r}\left(1-\left|r_{c}\right|\right)}\right),$$

where:

$$\rho_{r}=\left|\rho_{r}\right|e^{j\phi_{r}}=\sqrt{\frac{\langle {\left|S_{\mathrm{vv}}\right|}^{2}\rangle }{\langle {\left|S_{\mathrm{HH}}\right|}^{2}\rangle }}e^{j\left(\langle \phi_{\mathrm{vv}}-\phi_{\mathrm{HH}}\rangle \right)};$$

$$r_{c}=\frac{\langle S_{\mathrm{HH}}S_{\mathrm{VV}}^{*}\rangle }{\sqrt{\langle {\left|S_{\mathrm{HH}}\right|}^{2}\rangle \langle {\left|S_{\mathrm{VV}}\right|}^{2}\rangle }}.$$

It is observed that $\alpha_{B}$ is determined by two statistical measures, i.e., $\rho_{r}$ and $r_{c}$, which are the ratio of the averaged co-polarizations and the co-polarization coherence, respectively. In the second-order coherency matrix, both parameters can be obtained directly. However, it is difficult to use the parameter $\alpha_{B}$ to describe the scattering coherence. Thus, we introduce a parameter $\Delta \alpha_{B}$ to relate the effect of $r_{c}$, as follows:

(3) $$\Delta \alpha_{B}=\alpha_{B}-\alpha_{\mathrm{av}},$$

where:

(4) $$\alpha_{\mathrm{av}}=\tan^{-1}\left(\frac{{\left|\rho_{r}-1\right|}^{2}}{{\left|\rho_{r}+1\right|}^{2}}\right).$$

$\Delta \alpha_{B}$ can be used to measure the scattering randomness. Its sign is determined by the co-polarization phase difference (CPD). If all elemental scatterers in a resolution cell are consistent with one dominant scattering mechanism in both orientation and dielectric properties, where $r_{c}$ is large, then $\Delta \alpha_{B}$ is close to 0°; if scatterers vary considerably, where $r_{c}$ is small, then $\Delta \alpha_{B}$ is far away from 0°. For targets dominated by double-bounce scattering, since the physical model of double-bounce scattering is characterized by CPDs approaching to ±π [14], then according to (3), $\Delta \alpha_{B}$ should be smaller than 0°. In the next section, we analyze $\Delta \alpha_{B}$ and $\alpha_{B}$ for the reflection asymmetry case.

### 2.2. Parameters in the Reflection Asymmetric Case

Scatterers with non-reflection symmetric structures are often characterized by the polarization helicity τ. Helix scattering often occurs at places with complex man-made structures where double-bounce scattering is usually strong. In general cases, the existence of target helicity can add the detection of urban man-made buildings. When assuming that the scatterer has a significant target helicity, the corresponding coherency matrix can be shown as follows:

(5) $$T=Q{\left(2\theta \right)}^{T}\left[\begin{array}{ccc} t_{1}\cos^{2}\;2\tau & t_{3}\cos\;2\tau & \frac{j}{2}t_{1}\sin\;4\tau \\ t_{3}^{*}\cos\;2\tau & t_{2} & jt_{3}^{*}\sin\;2\tau \\ -\frac{j}{2}t_{1}^{*}\sin\;4\tau & -jt_{3}\sin\;2\tau & t_{1}\sin^{2}\;2\tau \end{array}\right]Q\left(2\theta \right),$$

where $t_{1}=\langle {\left|h+v\right|}^{2}\rangle /2$, $t_{2}=\langle {\left|h-v\right|}^{2}\rangle /2$, and $t_{3}=\langle \left(h+v\right){\left(h-v\right)}^{*}\rangle /2$. The effect of target orientation can be compensated by the target de-orientation procedure [15]. We assume the coherency matrix to be with the position of 0° orientation angle. By considering the scattering asymmetric parameter τ, the two parameters $\alpha_{B}$ and $\alpha_{\mathrm{av}}$ in (2) and (3) has the following forms, as shown in (6) and (7), respectively:

(6) $$\tan\left(\alpha_{B}\right)=\frac{T_{22}+T_{33}}{T_{11}}=\frac{\langle {\left|h-v\right|}^{2}\rangle }{\langle {\left|h+v\right|}^{2}\rangle }\frac{1}{\cos^{2}\;2\tau }+\tan^{2}\;2\tau$$

(7) $$\tan\left(\alpha_{\mathrm{av}}\right)=\left(\frac{{\left|\rho_{r}\left(\tau \right)-1\right|}^{2}}{{\left|\rho_{r}\left(\tau \right)+1\right|}^{2}}\right)$$

where:

$$\rho_{r}\left(\tau \right)=\sqrt{\frac{\langle {\left|S_{\mathrm{VV}}\left(\tau \right)\right|}^{2}\rangle }{\langle {\left|S_{\mathrm{HH}}\left(\tau \right)\right|}^{2}\rangle }}e^{j\left(angle\langle S_{\mathrm{VV}}\left(\tau \right)S_{\mathrm{HH}}^{*}\left(\tau \right)\rangle \right)},$$

$$\{\begin{array}{c} S_{\mathrm{VV}}\left(\tau \right)=-h\sin^{2}\;\tau +v\cos^{2}\;\tau \\ S_{\mathrm{HH}}\left(\tau \right)=h\cos^{2\;}\tau -v\sin^{2}\;\tau \end{array}.$$

Then, the difference between (6) and (7) can be used to analyse the effect of helicity τ on the parameter $\Delta \alpha_{B}$, as follows:

(8) $$\Delta \alpha_{B}=\alpha_{B}-\alpha_{\mathrm{av}}=\mathrm{atan}\left(\frac{\langle {\left|h-v\right|}^{2}\rangle }{\langle {\left|h+v\right|}^{2}\rangle }\frac{1}{\cos^{2\;}2\tau }+\tan^{2}\;2\tau \right)-\mathrm{atan}\left(\frac{{\left|\rho_{r}\left(\tau \right)-1\right|}^{2}}{{\left|\rho_{r}\left(\tau \right)+1\right|}^{2}}\right)$$

We can observe that $\Delta \alpha_{B}$ increases with τ. When backscatter is contributed by returns from helix type scatterers (reflection asymmetric scatterers), $\Delta \alpha_{B}$ is much closer to zero than those from reflection symmetric scatterers which are with the property of τ=0. For the symmetric case where τ=0, when r decrease, $\Delta \alpha_{B}$ approaches to −45°.

### 2.3. The $\Delta \alpha_{B}/\alpha_{B}$ Scattering Mechanism Classification Diagram

From the above analysis, it shows that for both helix and double-bounce type scatterers, $\Delta \alpha_{B}<0$ is always satisfied. We should note that the co-polarization ratio is affected by the target orientation angle, which would affect the scattering mechanism interpretation [15]. Thus before extracting $\rho_{r}$, a de-orientation procedure should be applied to rotate the scattering coherency matrix to be with 0° orientation angle [15].

**Figure 1:**
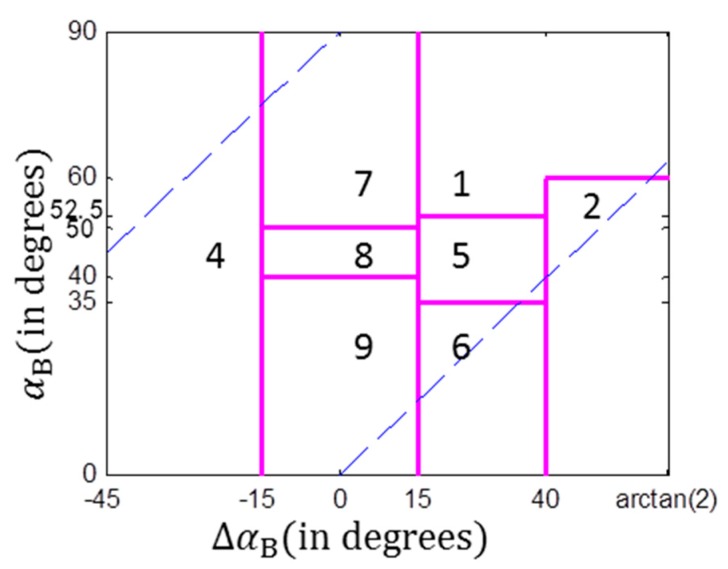
The $\Delta \alpha_{B}/\alpha_{B}$ scattering mechanism classification diagram, where the blue dash line indicates tilted boundaries. Zone 1: high entropy Multiple scattering; Zone 2: high entropy vegetation scattering; Zone 4: high entropy double-bounce scattering; Zone 5: medium entropy vegetation scattering; Zone 6: medium entropy dominant surface scattering; Zone 7: low entropy double-bounce scattering; Zone 8: low entropy dipole scattering; Zone 9: low entropy surface scattering.

$\alpha_{B}$ and $\Delta \alpha_{B}$ can be used to interpret the scattering mechanism and the scattering randomness, respectively, which have similar physical interpretations for target characterization as the polarization H and alpha. In [13], based on three scattering models and the alpha angle, a scattering segmentation plane was proposed as shown in Figure 1. By using this diagram, target physical scattering mechanisms can be classified into 8 classes. This diagram has similar interpretation for targets as the H/alpha plane. The main difference is that the H/alpha plane could not tell the difference between high entropy reflections from vegetated areas and urban areas, because both areas can generate multiple backscatters. The $\Delta \alpha_{B}/\alpha_{B}$ diagram integrates the phase difference to represent the scattering randomness. Two different zones, i.e., Zone 4 and Zone 2, are used to represent the multiple backscattering processes from the urban area and the vegetated area, respectively.

## 3. Experiments

**Figure 2:**
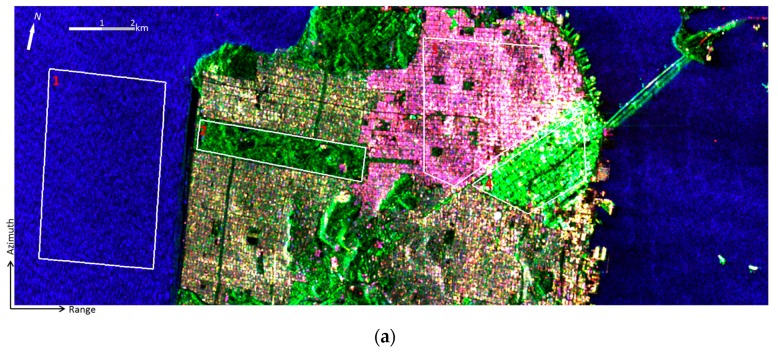

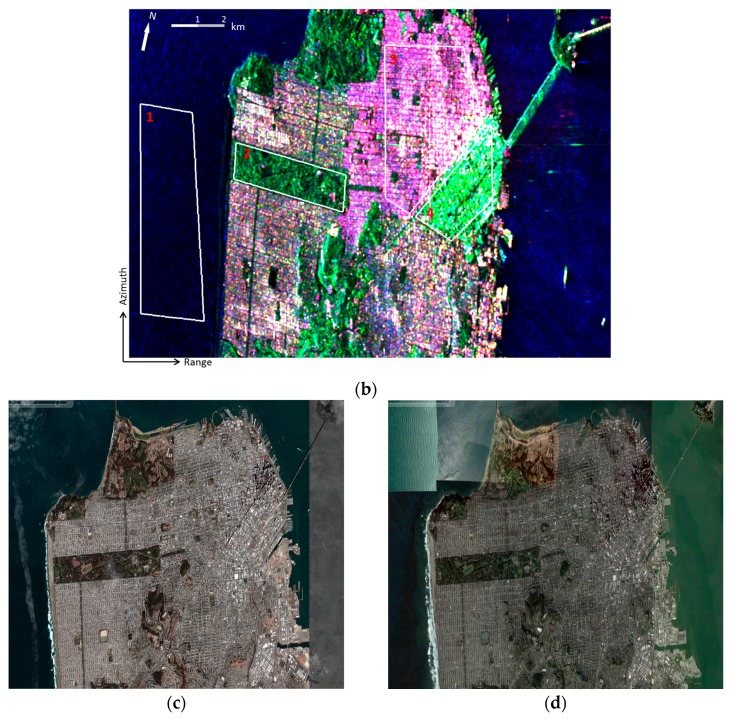
The Pauli-basis images of (**a**) GF-3 data collected on 15 September 2017, and (**b**) Radarsat-2 data collected on 9 April 2008. The outlined areas by white polygons are typical terrain types representing the ocean surface (1), forest (2), urban (3), and tilted urban (4) areas. (**c**) Google Earth image obtained on 25 September 2008, (**d**) Google Earth image obtained on 17 June 2017.

The San Francisco area in California (USA) was selected as the test site because this region has several typical terrain types, such as urban areas with different block directions, vegetated areas, and sea surface. Both GF-3 polarimetric SAR data and Radarsat-2 polarimetric SAR data acquired over this region are used for analysis. Radarsat-2 data is used as reference here, because the GF-3 sensor and the Radarsat-2 sensor both operate at C-band and have similar system parameters. Pauli-basis images and Google Earth images are shown in Figure 2. The GF-3 polarimetric SAR data was acquired on 15 September 2017 on ascending passes with right looking direction. The incidence angle ranges from 19.86 degrees to 22.59 degrees. The pixel space is about 5.37 × 2.25 m^2^. The image shown in Figure 2a has 1288 × 3250 pixels. The Radarsat-2 data was acquired on 9 April 2008 on ascending passes with right looking direction. The incidence angle ranges from 28.02 degrees to 29.82 degrees. The pixel space is about 4.73 × 4.82 m^2^. The image shown in Figure 2b has 1441 × 1988 pixels. Both data sets were filtered by a 7 × 7 sliding window.

Two kinds of experiments are carried out. First, the H/alpha method [6] and the $\Delta \alpha_{B}/\alpha_{B}$ method are used to analyze and evaluate the capability of GF-3 data for representing typical scattering mechanisms. Since the H/alpha method and the $\Delta \alpha_{B}/\alpha_{B}$ method have no relation with the total backscattered energy, in the second part of the experiments, the iterative Wishart classifier [16] is applied for further assessment based on the initial classified results obtained by the H/alpha and the $\Delta \alpha_{B}/\alpha_{B}$ diagrams. The outlined themes in Figure 2a,b is typical terrain types representing the urban, tilted urban, forest and ocean surface areas, which are used for quantitative assessment in the following experiments. In Figure 2, it is observed that there are two kinds of city blocks. The city block with buildings aligned along the azimuth direction is named as the urban area, and the city block with buildings/streets aligned approximately at 45 degrees off the azimuth direction is named as the tilted urban area. The tilted urban area often has specific orientation angles. We compared the Google Earth images of this area obtained in 2008 and 2017, and found that by visual inspection the terrain types in the selected areas were barely changed.

**Figure 3:**
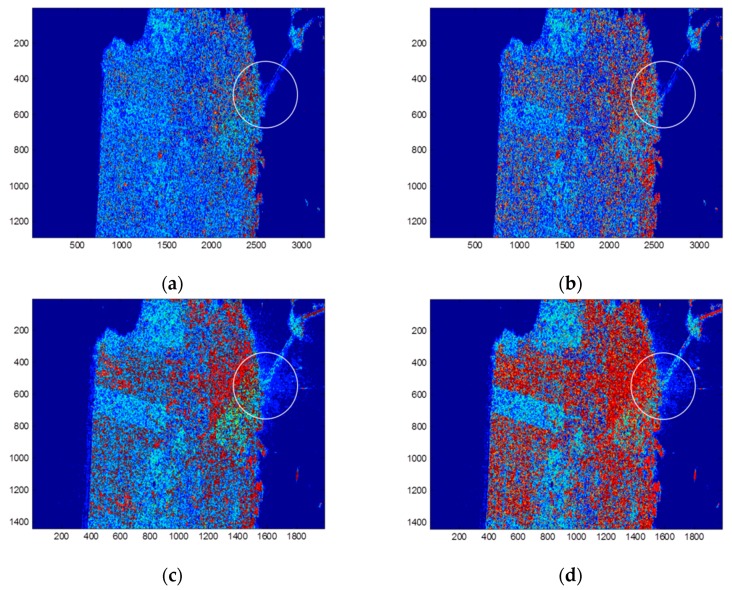
Physical scattering mechanism classification results by (**a**) the H/alpha method with GF-3 data; (**b**) the $\Delta \alpha_{B}/\alpha_{B}$ method with GF-3 data; (**c**) the H/alpha method with Radarsat-2 data; and (**d**) the $\Delta \alpha_{B}/\alpha_{B}$ method with Radarsat-2 data. The ocean surface in the white circle is easily affected by targets with strong backscatter on land.

The physical scattering mechanism classification results by the H/alpha and $\Delta \alpha_{B}/\alpha_{B}$ methods are shown in Figure 3. It is observed that by using the GF-3 data, both methods tends to classify less pixels to the classes dominated by double-bounce scattering, i.e., the red pixels in Figure 3a,b are less than those in Figure 3c,d. Compared with results of the Radarsat-2 data, results of the GF-3 data by using the H/alpha classification plane show little difference between the urban area and the forest area. This implies that the H/alpha classification plane may not be suitable for GF-3 image classification. By using the $\Delta \alpha_{B}/\alpha_{B}$ method, the capability of the GF-3 data for distinguishing different scattering types is improved.In the Radarsat-2 image, the ocean surface is classified into two scattering mechanisms, which is because the ocean surface area outlined in Figure 3 is highly affected by the adjacent double-bounce scattering, while in the GF-3 image, this area is not affected by the adjacent strong double-bounce backscatter. This may be due to the smaller incidence angle and the suppressing method used for sidelobes or ambiguities in GF-3 imaging.

**Figure 4:**
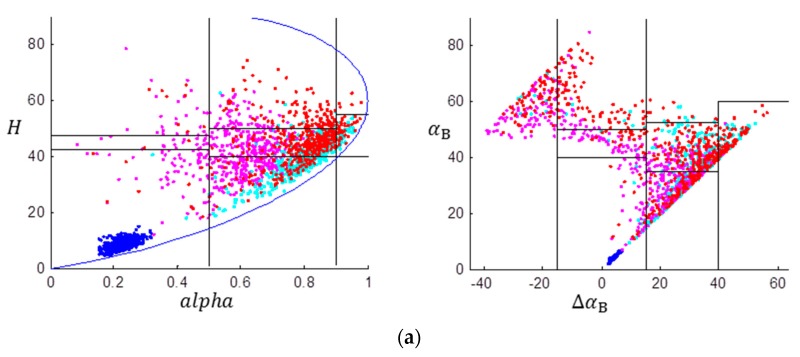

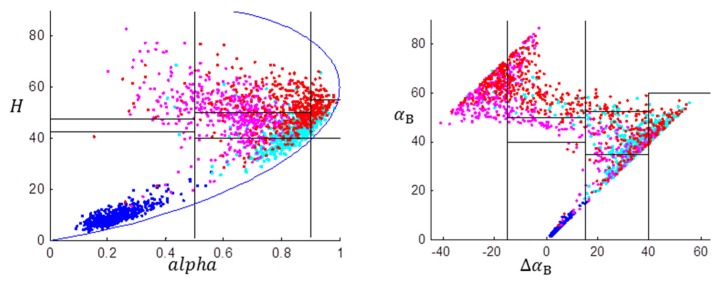
Scatter plots by using (**a**) the GF-3 data and (**b**) the Radarsat-2 data. Blue dots indicate samples from ocean surface, cyan samples from the forest area, magenta samples from the urban area, and red samples from the tilted urban area.

We randomly selected 500 samples from each typical themes and Figure 4 gives the H/alpha and the $\Delta \alpha_{B}/\alpha_{B}$ scatter plots. It is observed that by using the GF-3 data, samples from the ocean surface distributed more concentratedly than those of the Radarsat-2 data, and are more easily distinguished from the other theme pixels. This shows that the GF-3 data performs better in analyzing the physical scattering mechanism of ocean surface compared with the Radarsat-2 data. In both the H/alpha and $\Delta \alpha_{B}/\alpha_{B}$ diagrams, results of the Rasarsat-2 data shows that more pixels distribute in the zones with higher alpha and $\Delta \alpha_{B}$ values in comparison with the results of the GF-3 data. By using the outlined four typical terrain themes, Table 1 gives the percentages of the classified scattering mechanisms of each area. zi (i=1⋯9) denotes classification zone i (refer to Figure 1 for more details). The classification accuracy (CA) is calculated based on the physical interpretation of the predominant scattering mechanisms in each area. The CA for ocean is evaluated from the surface scattering, and thus pixels fall in zone 9 are used for the calculation of CA. Similarly, pixels in zone 5 and zone 2 are used to calculate the CA of forest, which is represented by the multiple vegetation scattering. Pixels in zone 7 and zone 4 are used to calculate the CA of the urban area, which is represented by the multiple even-bounce scattering. It is observed that the $\Delta \alpha_{B}/\alpha_{B}$ method provide higher overall classification accuracies for both data sets compared with the H/alpha method in identifying the predominant scattering types. By using the same method, the overall classification accuracy of the GF-3 data is not as good as that of the Radarsat-2 data. This implies that only the H/alpha plane or the $\Delta \alpha_{B}/\alpha_{B}$ diagram is not sufficient for GF-3 image classification.

Table 2 gives the statistical values of the physical parameters for the four typical areas. The mean value and the standard deviation are useful indicators for evaluating the between-class distance and with-in class deviation. For the purpose of classification, generally it is better to have a large between-class distance and a small with-in class deviation. We can observe that for the four themes, the discrimination abilities of parameter H of the GF-3 data and the Radarsat-2 data behave very similar. On the aspect of alpha images, the Radarsat-2 data shows a larger center difference between the forest area and the urban area in comparison with the GF-3 data, but both data sets have similar standard deviations in alpha. On the aspect of $\Delta \alpha_{B}$ and $\alpha_{B}$. parameters, the GF-3 data gives smaller standard deviations for the four themes than the Radarsat-2 data. However, it also bears a smaller distance between the forest and the urban theme centers. For the ocean area, the GF-3 data shows that the ocean theme is far away from the other theme centers with small standard deviations in all the four polarimetric parameters, further indicating that GF-3 data has a great potential for monitoring targets on the ocean surface.

The above analysis only considers the physical scattering mechanism of each terrain type. Next, we analyze the statistical property of the GF-3 polarimetric SAR data. The Wishart classifier [16] is a very classic classifier, which has been proved to be effective for many imaging scenarios and Earth observing missions.

**Figure 5:**
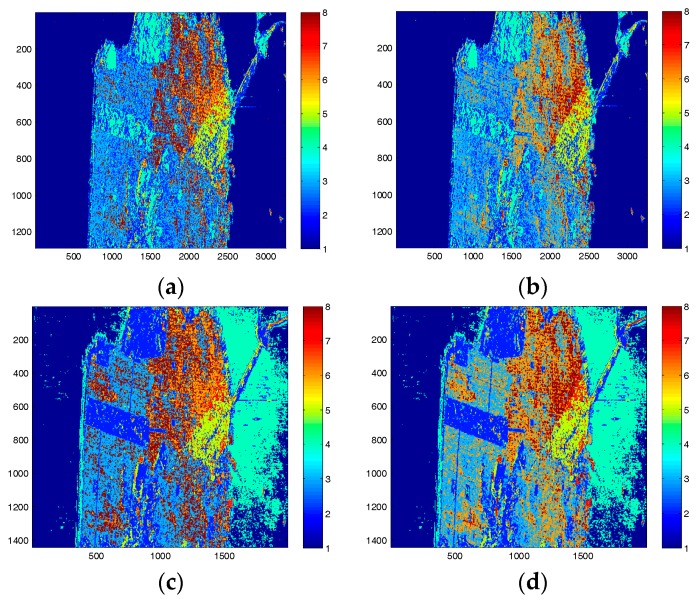
The Wishart classifier results initialized by the H/alpha plane are shown in (**a**,**c**), and results initialized by the $\Delta \alpha_{B}/\alpha_{B}$ diagram are shown in (**b**,**d**). (**a**,**b**) are results of the GF-3 data, and (**c**,**d**) are results of the Radarsat-2 data.

Before applying the iterative Wishart classifier, initial classification is needed. Both the H/alpha and $\Delta \alpha_{B}/\alpha_{B}$ classification results can be taken as initializations. Iteration of the Wishart classifier is set to stop when the total number of pixels switching between classes is less than 1% of the total number of pixels. The final results are shown in Figure 5. It is observed that results of the GF-3 data have a clear classified ocean surface. Since after the iteration pixels classified into class i(i=1…9). No longer correspond to the *i*-th zone of the scattering diagrams, we assume that in each typical scattering theme, the class with the maximum classified pixel number is selected as the correct classification labels for this area. After analyzing, we found that iterative results from zone 9 accounts for a significant number of pixels for ocean surface. Iterative results from zone 6 and zone 2 take up majority pixels for the forested area. Iterative results from zone 8 and zone 4 contribute a large proportion of pixels for the urban area, and iterative results from zone 1 accounts for majority pixels for the tilted urban area. By using this classification division, the confusion matrices for both GF-3 and Radarsat-2 data sets are shown in Table 3. It is observed that when applying the iterative Wishart classifier results of the GF-3 data and the Radarsat-2 data produce similar overall accuracies. This shows that when considering the statistical property in terrain classification, Radarsat-2 data and GF-3 data have similar performance. Since the two data sets were collected on different dates, this kind of small difference may be caused by disturbance of climate effects. For both data sets, the result initialized by the $\Delta \alpha_{B}/\alpha_{B}$. diagram slightly outperforms that by the H/alpha. initialization when the same convergence condition is applied.

## 4. Conclusions

In this study, the parameters $\alpha_{B}$ and $\Delta \alpha_{B}$, proposed based on the ratio of the co-polarization parameters under reflection symmetric case, was analyzed under the reflection asymmetry case. Then, both the $\Delta \alpha_{B}/\alpha_{B}$ diagram and the H/alpha method, together with the statistical Wishart classifier, are applied to analyze the performances of GF-3 polarimetric SAR data for target physical scattering mechanism interpretation and terrain classification. The $\Delta \alpha_{B}/\alpha_{B}$ diagram intrinsically integrates the co-polarization phase difference to discriminate between target scattering mechanisms and thus performs better than the classic H/alpha method. On average, the scattering mechanism identification accuracy of the $\Delta \alpha_{B}/\alpha_{B}$ method is about 3–5% higher than that of the H/alpha method by using the test data. The polarization entropy H shows similar performance with the GF-3 data and the Radarsat-2 data. However, the polarization alpha performs poorly with the GF-3 data. Results show that the $\Delta \alpha_{B}/\alpha_{B}$ diagram is effective in interpreting the physical scattering mechanism for GF-3 data. When using the classic Wishart classifier for terrain type classification, the GF-3 data and the Radarsat-2 data give similar classification accuracies. Further, GF-3 data shows its advantages for ocean surface monitoring. In future works, we will focus on the development of the statistical model-based classifier such that the GF-3 data can be efficiently used.

## Figures and Tables

**Table 1:** Classification accuracies (in percentage) of the H/alpha method and the $\Delta \alpha_{B}/\alpha_{B}$ method for four typical regions. The four areas were outlined in Figure 2. z1−z9 are the segmentation zones in both the H/alpha and $\Delta \alpha_{B}/\alpha_{B}$ planes [13].

**(A) Results by Using the GF-3 Data**										
	**The H/alpha Method**									
**%**	z9	z6	z5	z2	z1	z8	z7	z4	**CA**	**Overall Accuracy**
Ocean	100	0	0	0	0	0	0	0	100	59.47
Forest	1.00	46.33	40.86	8.90	0.11	0.03	0.26	2.51	49.76	
Urban	12.32	27.93	38.20	0.04	0	5.07	4.96	11.48	16.44	
Tilted Urban	2.35	17.23	44.30	8.89	1.40	0.35	1.45	24.03	25.48	
	**The $\Delta \alpha_{B}/\alpha_{B}$ Method**									
**%**	z9	z6	z5	z2	z1	z8	z7	z4	**CA**	**Overall Accuracy**
Ocean	100	0	0	0	0	0	0	0	100	62.58
Forest	1.74	44.50	35.81	12.54	1.09	0.54	2.21	1.58	48.35	
Urban	8.06	30.85	22.54	0.95	0.02	13.03	5.71	18.84	24.55	
Tilted Urban	2.96	16.78	32.10	11.29	2.77	2.39	17.32	14.40	31.72	
**(B) Results by Using the Radarsat-2 Data**										
	**The H/alpha Method**									
**%**	z9	z6	z5	z2	z1	z8	z7	z4	**CA**	**Overall Accuracy**
Ocean	99.87	0.13	0	0	0	0	0	0	99.87	69.90
Forest	0.17	29.28	49.95	14.94	0.35	0.08	0.30	4.93	64.89	
Urban	1.62	10.67	35.65	0.22	0.04	1.03	14.30	36.45	50.76	
Tilted Urban	0.83	4.81	26.70	16.54	7.18	0.23	3.19	40.51	43.71	
	**The $\Delta \alpha_{B}/\alpha_{B}$ Method**									
**%**	z9	z6	z5	z2	z1	z8	z7	z4	**CA**	**Overall Accuracy**
Ocean	99.93	0.07	0	0	0	0	0	0	99.93	75.22
Forest	0.31	28.40	42.38	19.20	1.93	0.67	3.88	3.24	61.59	
Urban	2.04	10.15	14.92	1.59	0.07	9.74	18.90	42.59	61.49	
Tilted Urban	1.06	4.66	18.45	11.62	5.83	1.32	32.49	24.59	57.07	

**Table 2:** The averaged values of different parameters of the four selected typical theme areas, where u denotes the averaged value and σ denotes the standard deviation.

u±σ		Ocean	Forest	Urban	Tilted Urban
H	The GF-3 data	0.22 ± 0.00	0.80 ± 0.01	0.57 ± 0.02	0.78 ± 0.02
	The Radarsat-2 data	0.23 ± 0.00	0.83 ± 0.01	0.62 ± 0.02	0.81 ± 0.02
alpha(°)	The GF-3 data	8.77 ± 1.94	40.07 ± 6.78	42.25 ± 8.37	46.46 ± 8.40
	The Radarsat-2 data	9.93 ± 3.06	42.94 ± 5.87	50.00 ± 9.43	51.87 ± 7.67
$\Delta \alpha_{B}\left({}^{\circ}\right)$	The GF-3 data	3.95 ± 0.93	29.60 ± 11.36	9.82 ± 20.55	16.72 ± 22.28
	The Radarsat-2 data	4.00 ± 1.84	30.56 ± 14.20	-3.82 ± 22.00	7.06 ± 24.42
$\alpha_{B}\left({}^{\circ}\right)$	The GF-3 data	4.19 ± 0.97	36.22 ± 8.84	41.20 ± 12.25	46.60 ± 12.41
	The Radarsat-2 data	4.39 ± 1.93	40.41 ± 9.32	52.77 ± 14.39	55.75 ± 12.29

**Table 3:** Comparison of the GF-3 and the Radarsat-2 data classification results by using the Wishart classifier.

	%	Ocean	Forest	Urban	Tilted Urban	Others	Overall Accuracy
(GF-3 data) H/alpha-Wishart	Ocean	100	0	0	0	0	84.29
	Forest	0.25	96.48	0.90	1.08	1.29	
	Urban	0.01	7.01	66.92	0.40	25.66	
	Tilted urban	0	29.33	3.04	58.53	9.11	
(GF-3 data) $\Delta \alpha_{B}/\alpha_{B}$-Wishart	Water	100	0	0	0	0	85.23
	Field	0.25	96.47	0.91	1.09	1.28	
	Forest	0.01	7.00	67.10	0.40	25.49	
	Urban	0	27.27	3.04	60.56	9.13	
(Radarsat-2 data) H/alpha-Wishart	Water	99.16	0	0	0	0.84	84.98
	Field	0.07	88.96	1.01	4.31	5.65	
	Forest	0	2.42	75.96	0.76	20.28	
	Urban	0	14.06	3.51	66.14	16.29	
(Radarsat-2 data) $\Delta \alpha_{B}/\alpha_{B}$-Wishart	Water	99.18	0	0	0	0.82	85.27
	Field	0.07	88.65	1.04	4.31	5.92	
	Forest	0	2.32	76.82	0.76	20.11	
	Urban	0	13.83	3.54	66.17	16.46	
